# Evaluation and SNP typing of DNA from ultraviolet-irradiated human bloodstains using TaqMan assay

**DOI:** 10.1038/s41598-021-87313-9

**Published:** 2021-04-13

**Authors:** Jian Tie, Seisaku Uchigasaki, Eiji Isobe

**Affiliations:** Division of Legal Medicine, Department of Social Medicine, Nihon University School of Medicine, Tokyo, 1738610 Japan

**Keywords:** Assay systems, Nanoscale materials

## Abstract

When detecting DNA profiles from forensic materials, it is pivotal to know the extent of degradation and which DNA marker can be genotyped. Ultraviolet (UV) is one of the common external factors that causes DNA damage, through which, an attempt to reveal cardinal genetic information can be made. In this study, after irradiation with three different UV wavelengths, UV-damaged DNA in the bloodstains was analyzed with long and short TaqMan assays using real-time PCR. In addition, both short tandem repeat (STR) profiles and single nucleotide polymorphisms (SNPs) from the damaged DNA at different stages of UV exposure were also assessed. With increasing in UV irradiation cycles, there was a delay of the amplification curves accompanied with a decrease in the DNA amounts collected. Despite the amplification of STR genotype was not altered after 75 cycles of UVC irradiation, all 12 SNP loci could still be detected. Furthermore, a short-assay line was detected in the absence of an amplification of the evaluation curve. The results indicate that, although the DNA template might not be useful and suitable for analysis of STR profile, this approach is of some values in detecting SNPs.

## Introduction

Retrieving evidence of genetic information from the degraded DNA samples is an important approach to reveal an individual identification in forensic medicine. Short tandem repeat (STR) polymorphisms are commonly used for the forensic identification of individuals^1^. However, DNA degradation is a major issue in this process. Various environmental factors (e.g., temperature, moisture, ultraviolet [UV] rays, and sample storage conditions) can lead to DNA hydrolysis or oxidization which can affect the results of tests. Amplification of STR profiles is difficult in cases in which the DNA is degraded, fragmented, and/or present in only very small amounts^2^. Some approaches have been suggested to improve the results of low copy number (LCN) DNA template analysis, such as optimizing DNA extraction methods, increasing the number of cycles of PCR, repairing degraded DNA specimens, and using whole-genome amplification (WGA)^3–5^. However, the use of these methods in real forensic applications is limited, despite the fact that it is often necessary to produce DNA profiles from degraded LCN forensic materials. Although STRs exhibit high discriminability, their use is limited by their high mutation rates, the requirement for large amplicon sizes, and the existence of artifacts in practical applications, all of which can have negative influence on downstream analysis^6^. Single nucleotide polymorphisms (SNPs) have recently been introduced to identification protocol to overcome these limitations^7^. The SNP is defined as a change in a single nucleotide that is present in more than 1% of the general population. The SNP analysis can be used for various genetics studies.

Nearly all existing criminal DNA databases consist of STR profiles. There is a legacy issue with this, in that SNP profiles cannot be compared with STR profiles. It is possible, however, that SNPs will play a more important role in forensic identification in the future. Many studies have reported that SNPs have some advantages over STRs in the forensic identification of individuals and paternity testing^8,9^, for example, the amplicons involved in SNP analysis are very small; so they can used for analysis of highly degraded samples^10^. In addition, the SNP mutation rate is relatively low, which is advantageous in kinship analysis^11^.

Autosomal SNPs are appropriate individual genetic markers because of their low mutation rates, distribution across the whole genome, and stability in genetic analysis of races. Additionally, databases of the allele frequency patterns of most people can be compiled as only small amplicons are required for SNP analysis and high-throughput genotyping technology is effective. Blood is a form of biological evidence that is commonly found at crime scenes^12^. Important genetic information can be obtained from a bloodstain, which aids in the identification of victims and suspects during forensic analysis. However, bloodstains at crime scenes are often small and thus often remain undiscovered for prolonged periods, during which the DNA deteriorates. Even if the degraded DNA can be amplified by PCR, the results are affected by any decrease in the quantity of DNA.

When detecting DNA profiles from forensic materials, is necessary to know the extent of DNA degradation and which DNA marker can be genotyped in these samples. In forensic DNA identification, no template found for investigators and researchers to use for analysis, it is thus important to first select a suitable method that is sensitive to the degraded DNA specimens which are usually small in quantity. The UV is one of common external factors that break down DNA molecules, and human bloodstains are also one of the biological samples that are commonly used for DNA analysis in forensic caseworks. However, reports on the status of degradation of UV-damaged DNA samples and the investigation of the relevant genetic markers from these samples are scant^13,14^. In the present study, after irradiation with three different UV wavelengths, UV-damaged DNA in the bloodstains was analyzed with long and short TaqMan assays using real-time PCR. Furthermore, both STR profiles and SNPs from the damaged DNA at different stages of UV exposure were also assessed.

## Results and discussion

### DNA degradation by UV irradiation

Analysis of DNA fragmentation by real-time PCR with long and short TaqMan assays showed a change in DNA degradation after each UV irradiation cycle (data not shown). After 15 cycles of UV irradiation, long-assay amplification gradually became difficult, UVA irradiation caused the most issues, followed by UVB irradiation, and finally UVC-irradiation. Conversely, we observed no notable difference in short-assay amplification between UVA- and UVB-irradiated dried blood samples, although amplification was slow from UVC-irradiated dried blood samples. When the amount of UV irradiation was increased to 75 cycles, short-assay amplification became more difficult, but amplification curve could still be observed for all UVA-, UVB-, and UVC-irradiated dried blood samples. However, in long assay amplification, the amplification cycle was delayed compared to short-assay amplification. Also, no long assay amplification curve was observed for UVC-irradiated dried blood samples.

In addition, we found that the quantity of DNA collected from the dried blood samples decreased with an increase in the number of UV irradiation cycles compared to DNA from samples that had not been exposed to UV irradiation. After 45 cycles of irradiation, the quantity of DNA collected from UVC-irradiated dried blood samples was less than half of that collected from UVB-irradiated dried blood samples and even less of that collected from UVA-irradiated dried blood samples. Some methods of DNA quantification have already been reported; however, the method reported in the present study allows for the evaluation of a fragment of DNA in addition to quantification. Furthermore, the procedure is simple.

The results of analysis by two methods, TaqMan and Quantifiler Trio, were not significantly different in terms of the quantity of DNA (*p* > 0.05). In addition, the substrates on which DNA samples were placed to mimic crime scene sample did not affect either analysis. Furthermore, the quality and quantity of DNA was not significantly different between bloodstains on glass slides and on cotton gauze stored indoors in darkness (p > 0.05).

### Amplification of STR profiles

**Figure 1:**
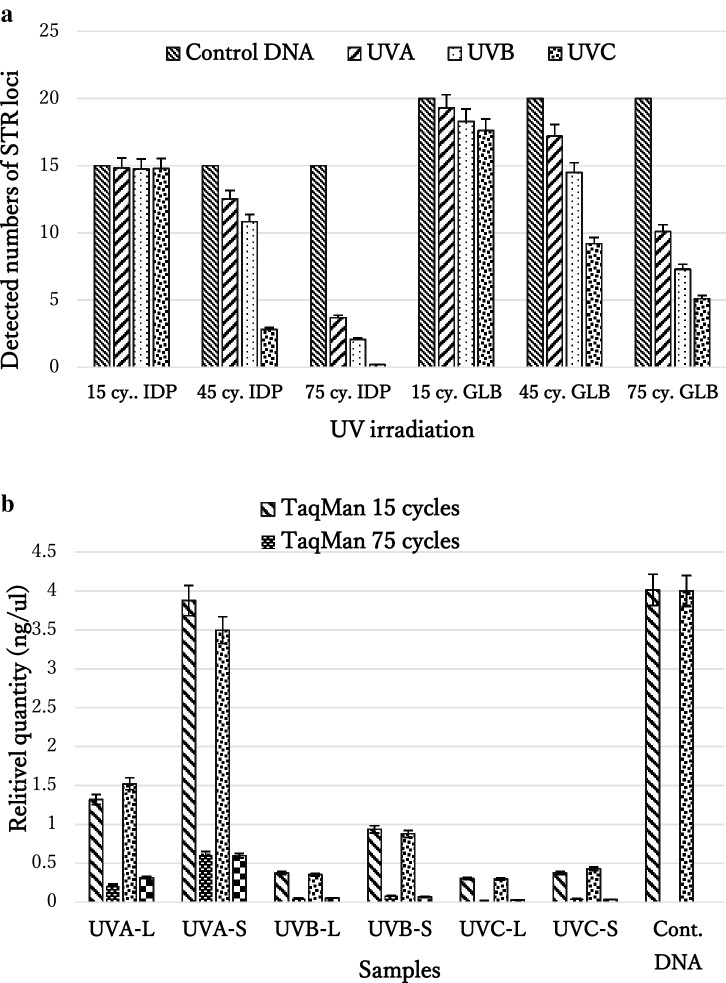
(**a**) Average number of detected STR loci ion human bloodstains using IDP and GLB methods following UV irradiation in each three cycles (15, 45 and 75). (**b**) Quantity of DNA **in** dried blood samples detected by long (-L) and short (-S) TaqMan assays as well as large autosomal (-L) and small autosomal (-S) Quantifiler Trio using real-time PCR.

In the present study, we attempted to irradiate dried blood samples with higher UV intensities than in a previous study^15^. STR genotypes were more difficult to detect after fewer irradiation cycles, a full STR profile could not be obtained even after 45 cycles of UVA irradiation. When the number of UVA, UVB, and UVC irradiation cycles was increased to 75, we were able to confirm three STR loci for all samples. The GlobalFiler PCR Amplification kit (GLB) shares 14 STR loci with the AmpFlSTR Identifiler Plus Kit (IDP), The results of these two methods were compared, and it was found that the GLB method detected more STR loci than the IDP method, but no significant difference was found in the 14 shared loci after each cycle of UVA irradiation (*p* > 0.05). However, the number of STR loci detected by the GLB method was markedly higher than the number detected by the IDP method in samples irradiated for 45 and 75 cycles (*p* < 0.05). Although the number of STR loci detected by the GLB method increased universally, the number of STR loci detected in bloodstains irradiated with 75 cycles of UVC remained at an average of five. After prolonged UV-irradiation, the DNA showed severe degradation, making it difficult to amplify to detect STRs. (Fig. 1a).

At least the following three artifacts often occur in the detection of STR profile from LCN materials: One is the drop-in of alleles. This is often due to sample contamination. The second is the allele dropout. This occurs due to sample degradation. The third is the appearance of stutter peaks. It is commonly observed in 5–10% of alleles. Based on these facts, STR is considered to have high uncertainty in LCN samples. In this study, even if contamination was avoided, but the allelic dropouts and stutter peaks still were observed when amplification of STR profiles from UV irradiated bloodstain DNA.

### SNP genotyping

**Figure 2:**
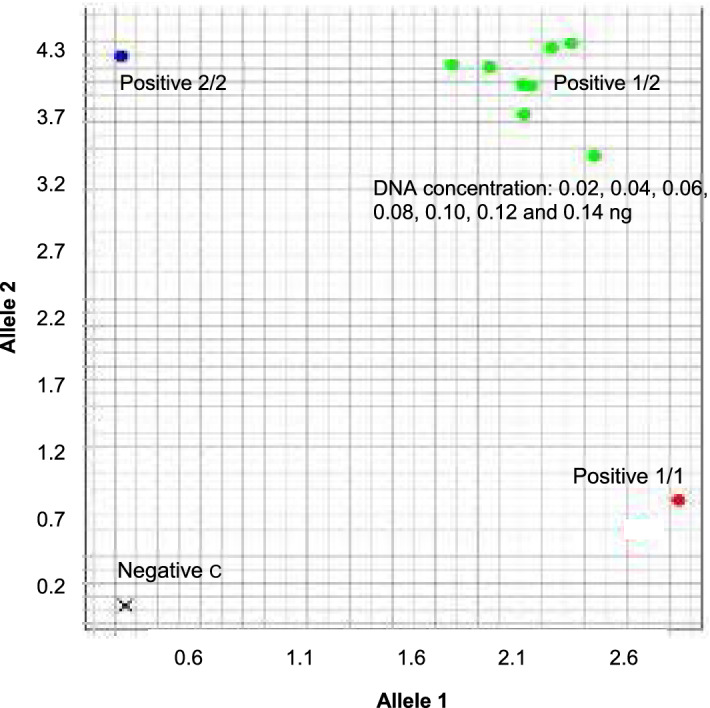
Detectability of DNA template with TaqMan assays. For DNA extracted from dried blood samples, the amplified SNP locus was rs1528460, and the positive DNA control used 0.06 ng of the DNA template for real-time PCR amplification.

**Figure 3:**
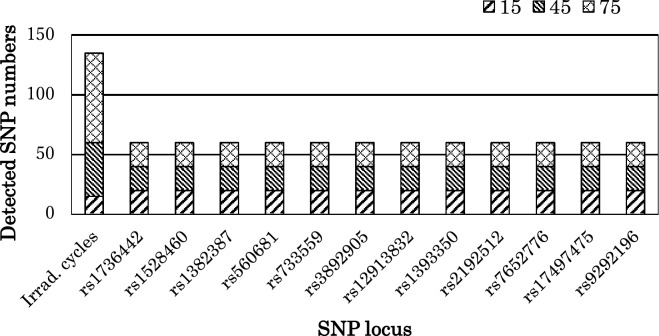
Genotyping of SNPs amplified from UV irradiated bloodstains from 20 bloodstain samples.

All of the 12 SNP loci were detected in non-irradiated dried blood samples, which confirmed the sensitivity of this method of using diluted DNA at different concentrations (0.02–0.14 ng). The results indicated that each allele in each diluted template was amplified at all concentrations and that 0.02 ng of DNA template was required for SNP genotyping in the TaqMan assays in the present study (Fig. 2). In comparison with the SNPs of non-irradiated bloodstains, the SNPs of irradiated bloodstains showed good reproducibility. Therefore, although fewer than three STR loci were undetectable using the IDP method and fewer than five STR loci were undetectable using the GLB method after 75 cycles of UV irradiation of 20 dried blood samples, 12 SNP loci were genotyped by TaqMan assays. The results indicated that although the dried blood samples were treated with longer cycles of UV light and the quality and quantity of the irradiated DNA decreased (Fig. 1b), the SNP genotypes of those 12 SNP loci could still be successfully amplified (Fig. 3).

For exact amplification of the DNA genotype of forensic samples, not only the quantity of DNA available but also its quality matter. As PCR technology progresses, detection of DNA profiles is improving. In addition, there are some methods such as agarose gel electrophoresis or pulsed-field gel electrophoresis that can be applied to separate larger DNA fragments^16^. Real-time PCR is a simple, sensitive method of evaluating degraded DNA specimens, and only requires a very small amount of DNA. The analysis is based on a calibration curve of standard DNA and long and short TaqMan assays, and after comparing the amplification results, is easy to discern the extent of DNA fragmentation and determine whether the DNA is suitable for polymorphism amplification.

UV light causes degradation of the DNA in biological samples^17^. In the present study, to evaluate degraded forensic samples, we simulated the destruction of DNA in human bloodstains by irradiation with different UV wavelengths. Amplified TaqMan assays plots after cycles of irradiation with three wavelengths showed the extent of DNA degradation. The strength of irradiation highest with UVC, followed by UVB, then finally UVA. We reinforced the UV irradiation energy to shorten the UV irradiation period in this study. After analysis of DNA fragmentation by TaqMan assays, STR and SNP profiles were determined after every 15 cycles of UV irradiation. To consistently generate all 15 of STR loci using the IDP PCR amplification Kit, at least 0.125 ng of DNA template is needed^18^. However, as the number of DNA lesions increases, the quantity of DNA collected decreases and genotyping becomes difficult. In the present study, no full STR profiles were observed for UVC- and UVB-irradiated dried blood samples after 45 cycles and for UVA-irradiated dried blood samples after 45 cycles. It is common knowledge that for amplification of DNA polymorphisms from LCN forensic DNA samples, SNPs are more efficient than STRs because SNPs can be detected from short fragment of the DNA template^19^.

In the present study, we attempted to amplify 12 SNP loci from DNA from UV-irradiated dried blood samples using TaqMan assays. The results indicated that only 0.02 ng of DNA template are needed to detect one locus of the SNP genotype. After 75 cycles of UVC irradiation, DNA amplification did not show the STR genotype; however, all SNP loci could still be detected. Even the evaluation curve of amplification by real-time PCR was not amplified and only showed a short-assay line, indicating that the DNA template could not be analyzed for STR profiles, but it might be possible to detect SNPs. In other words, when the long assay of a sample cannot be amplified, the DNA template might be inappropriate for STR profile amplification.

Various SNP analysis methods have been reported previously; however, in the present study, we paid more attention to question of how to simply detect SNPs in damaged DNA samples. The SNP loci studied in the present study are commonly used in our lab, all 12 SNPs have been described in several populations, none of which show significant deviations from Hardy–Weinberg equilibrium ratios or strong linkage and all of which have a heterozygosity of over 0.31^20–24^. We attempted to use only 12 SNPs genotypes detection and comparison with STRs. The 52-plex system is more powerful for individual identification, however, this was detection by a basic study, which determines when the STRs could not play for the damaged samples, and we have another choices like SNPs to analyze those samples. Furthermore, the TaqMan assay method that was used in this study was a single PCR amplification; therefore, it was easy to determine how many SNPs could be amplified or which SNP locus was difficult to amplify when the results.

Although several approaches have been proposed to improve the results of analysis of LCN DNA templates for genetic markers, including the WGA method^25^, at least 0.1 ng of DNA template is needed for STR profile amplification. Moreover, before STR profile amplification, the WGA products need to be purified. Therefore, SNP locus amplification may be an appropriate choice in cases where STR profile amplification cannot be used to analyze LCN forensic samples.

## Materials and methods

### Material

As a UV irradiation sample, we collected blood samples from 20 Japanese subjects by venipuncture. The blood samples were spotted onto sterile cotton gauze and allowed to dry at room temperature. To mimic bloodstains recovered from a crime scene, about 1.5 ml of the blood was deposited onto glass slides (7.6 × 2.6 cm), pieces of wood (3 × 2 × 1 cm), disposable conical tube caps (1.5 × 1.3 cm), a metal Japanese coin (10 yen), disposable nitrile gloves and pieces of fabric. In order to ensure reproducibility, all bloodstains were divided into three equal parts for used in later experiments.

### UV irradiation of blood samples

We exposed the dried blood samples to UVA (365 nm), UVB (302 nm), and UVC (254 nm) light in a UV cross-linker (Ultra Violet Products, Upland, CA, USA) at an intensity of 120 mJ/cm^2^ under a 16 h light/3 h dark cycle. We repeated the UV irradiation for 75 cycles until no more than three STR loci were detectable by IDP amplification. All samples were then stored in the dark until PCR amplification.

Two control bloodstains with the same amount blood were used, one was on a glass slides and the other was on sterile cotton gauze. These samples were kept in darkness for the same a length of time with UV treated bloodstains.

### DNA isolation of bloodstains

The cotton gauze with dried blood samples was cut into 1.5 cm^2^ squares, and bloodstains on the materials were transferred to gauze. Then DNA extraction was performed using the QIAamp DNA Blood Investigator Kit (QIAGEN Inc., Valencia, CA, USA). The isolated DNA samples were prepared for quantification using the Qubit dsDNA HS Assay Kit (Life Technologies, Waltham, MA USA) and then quantified using the Qubit 3.0 Fluorimeter (Life Technologies) according to the manufacturer’s instructions.

### Evaluation of UV irradiated DNA fragmentation

To understand the progress of DNA degradation in detail, we extracted 2 μL of DNA solution from the dried blood samples and analyzed DNA fragmentation by real-time PCR using the StepOnePlus Real-Time PCR System (Applied Biosystems, Foster City, CA, USA). The real-time PCR reaction mixture contained 10 µL TaqMan Universal Master Mix II (Applied Biosystems), 1 µL TaqMan assay, and 7 µL nuclease-free water. TaqMan MGB Gene Expression Kits containing a long assay (256 bp) and TaqMan RNase P Detection Reagents as a short assay (87 bp) were used for DNA fragmentation analysis. A FAM-TAMURA-labeled probe was used, and the Human Control DNA set attached to the kit was used to make a calibration curve for each analysis plate. The primers and probes were as follows: long assay forward primer 5′-CCCTTGGGAAGGTCTGAGACTAG,3′, reverse primer -5′-CGGAGGAGAGTAGTCTGAATTGG′,3, TaqMan MGB probe -5′-CTCACCTCAGCCATTG′,3, The short assay detected the ribonuclease P RNA component H1 (H1RNA) gene (RPPH1) on chromosome 14, and cytoband 14q11.2. The assay location was chr.14:20811565 on NCBI build 37. It had an 87 bp amplicon that mapped to a single exon RPPH1 gene. The real-time PCR thermal cycling protocol was 50 °C for 2 min and 95 °C for 10 min, followed by 45 cycles of 95 °C for 15 s and 60 °C for 60 s.

A standard Quantifiler Trio DNA Quantity Kit (Applied Biosystems) was used for comparison of DNA quantity and quality with a TaqMan assay of 15 male samples. The analysis was performed with QuantStudio 5 (Applied Biosystems) according to the instructions in the user guide.

### STR analysis

All 15 autosomal STR loci (D8S1179, D2S11, D7S820, CSF1PO, D3S1358, TH01, D13S317, D16S539, D2S1338, D19S433, vWA, TPOX, D18S51, D5S818, and FGA) and amelogenin were amplified by PCR from 1 ng DNA isolated from dried blood samples using the AmpFISTR Identifiler Plus (IDP) PCR Amplification Kit (Applied Biosystems) according to the manufacturer’s instructions. The PCR-amplified DNA fragments were detected using the ABI Prism 310 Capillary Electrophoresis System (Applied Biosystems). STR analysis was performed on DNA isolated from dried blood samples before and after UV irradiation for comparison of STR profiles. DNA detection was performed after every 15 cycles of UV irradiation. Furthermore, a GLB PCR Amplification Kit with 21 STR loci was used to amplify every sample twice for comparison of STR detection using an Applied Biosystems 3500 Genetic Analyzer.

### SNP genotyping

After several cycles of UV irradiation, examination of STR profiles from the dried blood samples became difficult, and we tried to perform SNP genotyping instead. We performed 12 TaqMan SNP Genotyping Assays (Thermo Scientific; Table 1) using the StepOnePlus Real-Time PCR System in a reaction volume of 10µL, which comprised 2× TaqPath ProAmp Master Mix (Applied Biosystems), 0.25 µL 40× TaqMan SNP Genotyping Assay (containing primers and probes), and 1.0 µL genomic DNA samples. The genotyping amplification protocol was 60 °C for 30 s and 95 °C for 5 min, followed by 45 cycles of 95 °C for 5 s and 60 °C for 30 s. To confirm the detection, multiple diluted standard DNAs (0.02 ng to 0.14 ng) were used to detect the SNP genotypes. As controls, each SNP was detected in all bloodstains before UV irradiation. Detailed information on the SNPs that were used in this study is deposited in dbSNP.

**Table 1:** SNP loci for analysis of ultraviolet-irradiated dried blood samples.

No	SNP ID	Allele	Chromosome	MAF
1	rs1736442	C/T	Chr18	0.3569
2	rs1528460	C/T	Chr.15	0.2752
3	rs1382387	A/C	Chr.16	0.2946
4	rs560681	A/G	Chr.1	0.2926
5	rs733559	C/T	Chr.14	0.2897
6	rs3892905	A/G	Chr.3	0.2770
7	rs12913832	A/G	Chr.15	0.0830
8	rs1393350	A/G	Chr.11	0.0793
9	rs2192512	C/T	Chr.20	0.4530
10	rs7652776	C/G	Chr.3	0.4645
11	rs17497475	A/C	Chr.4	0.4792
12	rs9292196	C/T	Chr.5	0.1542

*MAF* minor allele frequency.

### Statistical analysis

Data from the TaqMan assay, Quantifiler Trio and the STRs were tested using one-way ANOVA analysis for statistical significance.

### Compliance with ethical standards

Ethical approval for this study was granted by the Ethics Committee of Nihon University School of Medicine and National Research Committee. All procedures performed in studies involving human participants were in accordance with the ethical standards of this Ethics Committee and with the 1964 Helsinki declaration and its later amendments or comparable ethical standards. Informed consent was obtained from all participants included in the study.
